# FT-ICR-MS reveals the molecular imprints of the brewing process

**DOI:** 10.3389/fnut.2023.1243503

**Published:** 2023-09-22

**Authors:** Stefan A. Pieczonka, Martin Zarnkow, Friedrich Ampenberger, Martina Gastl, Michael Rychlik, Philippe Schmitt-Kopplin

**Affiliations:** ^1^Analytical Food Chemistry, TUM School of Life Sciences, Technical University of Munich, Freising, Germany; ^2^Analytical BioGeoChemistry, Helmholtz Association, Helmholtz Munich, Neuherberg, Germany; ^3^Research Center Weihenstephan for Brewing and Food Quality, Technical University of Munich, Freising, Germany

**Keywords:** beer, brewing process, FT-ICR-MS, multivariate statistics, metabolomics, molecular profiles, Maillard reaction

## Abstract

The study of fermentation and brewing has a long history of pioneering discoveries that continue to influence modern industrial food production. Since then, numerous research endeavors have yielded conventional criteria that guide contemporary brewing practices. However, the intricate open challenges faced today necessitate a more exhaustive understanding of the process at the molecular scale. We have developed an ultra-high-resolution mass spectrometric analysis (FT-ICR-MS) of the brewing process that can rapidly and comprehensively resolve thousands of molecules. This approach allows us to track molecular fluctuation during brewing at the level of chemical compositions. Employing biological triplicates, our investigation of two brewing lines that are otherwise identical except for the malt used revealed over 8,000 molecular descriptors of the brewing process. Metabolite imprints of both the similarities and differences arising from deviating malting temperatures were visualized. Additionally, we translated traditional brewing attributes such as the EBC-value, free amino nitrogen, pH-value, and concentration curves of specific molecules, into highly correlative molecular patterns consisting of hundreds of metabolites. These in-depth molecular imprints provide a better understanding of the molecular circumstances leading to various changes throughout the brewing process. Such chemical maps go beyond the observation of traditional brewing attributes and are of great significance in the investigation strategies of current open challenges in brewing research. The molecular base of knowledge, along with advancements in technological and data integration schemes, can facilitate the efficient monitoring of brewing and other productions processes.

## 1. Introduction

The brewing of beer has accompanied humanity and its civilization for millennia (1). Fermented and thus durable beverages such as beer are one of the reasons for the domestication of grains and thus settling down. They played a significant role in shaping cultural practices and traditions around the world. From ancient ceremonies and rituals to modern-day social gatherings, the consumption of beer has been intertwined with human customs and traditions for centuries (2). However, the significance of beer extends far beyond cultural heritage. Brewing science and fermentation research have also been instrumental in driving technological advancements and innovations across a range of fields, including microbiology, food science and hygiene, and industrial food production. Pioneers such as Enzinger, Pasteur, Linde, and Hansen paved the way for modern science and new technologies that continue to shape our world today (3–6).

Since then, our understanding of the brewing process has improved significantly. Sophisticated brewing attributes that describe and guide the process have been established and standardized (7, 8). However, some challenges in brewing science remain unresolved. The complexity of brewing (9–12)—which borders on agriculture, combines the biology of raw materials with the biochemistry of fermentation and the chemistry of heat-induced or oxidative molecular changes—leading to convoluted issues that cannot be fully resolved through simplifying attributes.

Parallel to the evolution of brewing, new techniques and methodologies have been established in the field of analytical chemistry. Targeted univariate measurements are complemented by non-targeted approaches, which promise to reveal a large part of the molecular world of beer and make it accessible through rapid profiling measurements (10). Particularly, the continuously increasing resolution of mass spectrometry opens the possibility to analyze thousands of compounds simultaneously. Applying the resolution power of Fourier-transform ion cyclotron resonance mass spectrometry (FT-ICR-MS) has already shown how such holistic analytics can break new grounds to describe the diverse beer metabolome (13). Characterizing brewing methods (14), authenticity testing against the background of the German purity law (15), and the comprehensive analysis of the complex Maillard reaction network during brewing (16) benefit from the molecular mapping of beer.

Contrary to mentioned studies, the focus of the presented research is not on the bottled beer but on the entire brewing process from the grain to the finished beer. With the help of ultrahigh-resolution analytics, we aim to make the molecular diversity and complexity of the brewing process visible and establish deep chemical imprints for the decisive process steps. In addition, multivariate statistics [HCA, PCA, OPLS-(DA)] will be used to correlate the relationship of classical brewing attributes with molecular profiles comprising hundreds of metabolites. A molecular base of knowledge will be unveiled through a defined and reproducible experimental design. The investigations will focus on two brewing series that differ solely in the kilning temperature applied to the same barley, while maintaining uniform conditions for all other aspects of the brewhouse process. This approach enables the evaluation of the impact of Maillard reaction during kilning on the overall molecular composition of subsequent stages of the brewing process. Moreover, it provides a means to characterize the inherent molecular processes that remain consistent during brewing, independent of the influence of the Maillard reaction. We aim to demonstrate the potential of ultrahigh-resolution mass spectrometry to address the convoluted questions in brewing research and facilitate efficient monitoring and control of the brewing process in the future.

## 2. Materials and methods

### 2.1. Beer samples

**Figure 1:**
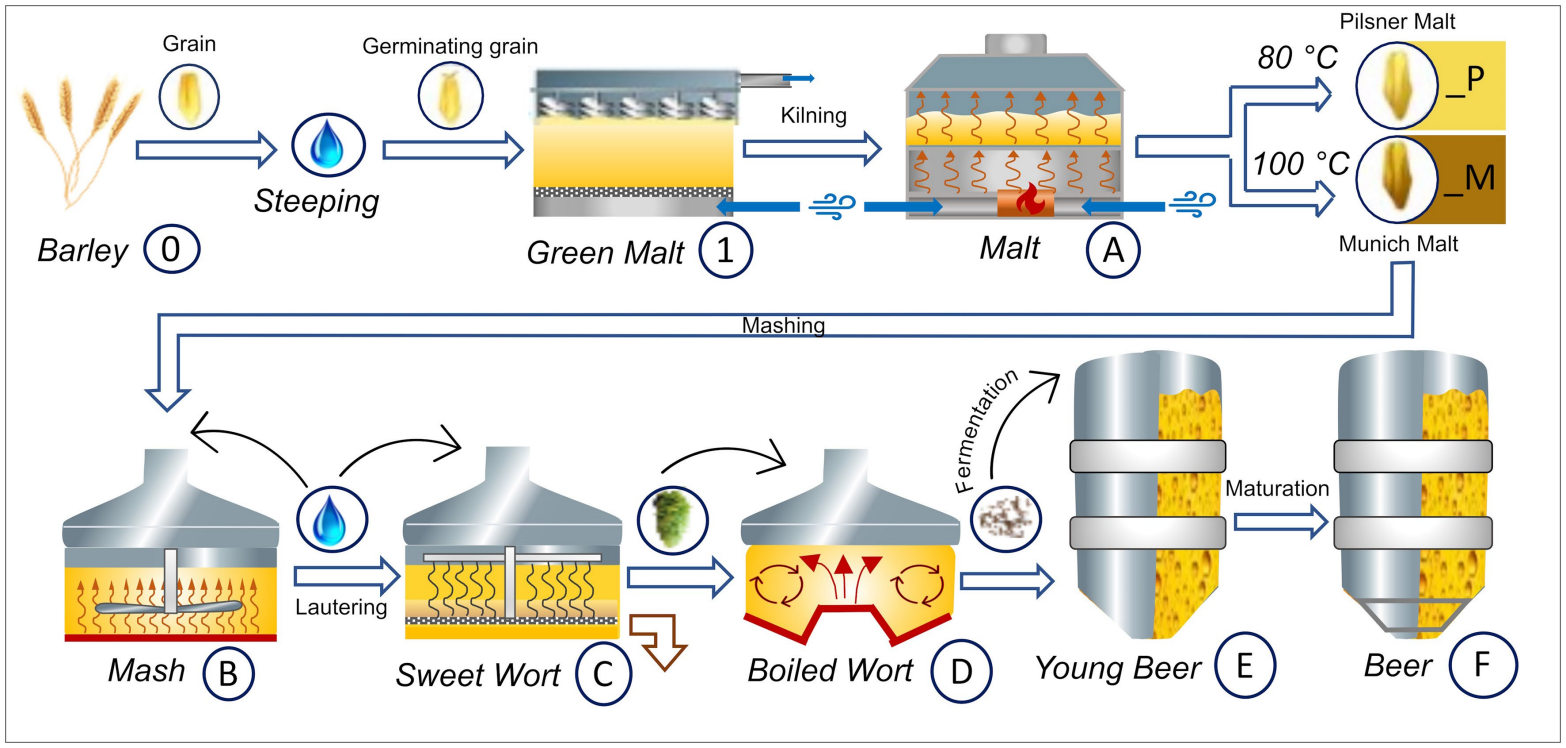
Graphical overview of the Brewing process carried out in triplicates and respective sampling points. The barley grain (0) is brought to germination resulting in green malt (1). The final malt (A) is obtained by kilning at 80°C for Pilsner malt (_P) and at 100°C for Munich malt (_M), respectively. Both malts are brewed in the same way, resulting in two sets of samples (_P and _M). After the mashing process (B_P and B_M) the beer gets lautered producing the sweet wort (C_P and C_M). After addition of hops, boiling and separation of the lees in the whirlpool, the boiled wort is obtained (D_P and D_M). Fermentation with *Sacc. pastorianus* gives the young beer (E_P and E_M) that, after maturation, is filtered and bottled (F_P and F_M). This figure has been provided by ©Simone Schinko with permission for publication under the Creative Commons Attribution License (CC BY).

The beers were brewed with *Hordeum vulgare* L. variety Accordine in biological triplicates of 25 liter for each sample series. One set of triplicates was carried out with Pilsner malt (indicated by a _P suffix) and the other with Munich malt (indicated by a _M suffix). A graphical overview of the experimental brewing process and sampling points—resulting in three biological replicates of samples 0 to F_P and 0 to F_M, respectively—is given in Figure 1. The following process steps were sampled: raw barley (0), green malt (1), malt (A_P and A_M), mash (B_P and B_M), sweet wort (C_P and C_M), boiled wort (D_P and D_M), young beer (E_P and E_M), and the finished beer (F_P and F_M), respectively. Detailed parameters of the brewing process can be found in Supplementary Table S1 and are summarized in the following. For each replicate, malting was carried out in eight 1 kg batches and 5.2 kg of the resulting malt was used for subsequent mashing. The Pilsner malt triplicates were kilned at 80°C, the Munich malt at 100°C for 5 h, respectively. Mashing resulted in gravity contents of 11.5 to 11.7 (M), and 10.6 to 11.7 (P) percent by weight, respectively. Before boiling for 60 min, 35 g of hops *Humulus lupulus* L. pellets variety Tradition (6.6% α-acids) was added. Fermentation was carried out for about 20 days at 12°C using SafLager™ TUM 34/70 *Saccharomyces pastorianus* dry yeast, resulting in an ethanol concentration by volume of 4.8–4.9 (M), and 4.7–4.9 (P), and an apparent degree of fermentation of 78.3–79.5% (M), and 82.9–83.5% (P), respectively. Maturation was considered complete at a diacetyl (2,3-butandion) content of less than 0.1 mg/L, and the beers with EBC values of 14.8–15.9 (M), and 5.9–6.3 (P) were subsequently bottled.

### 2.2. Beer attributes

The content of amino acids (LS-HPLC001 2018–07), free amino nitrogen [MEBAK Bd. WBBM 2012 (17) chapter 2.6.4.1.1], fermentable sugars (chapter 2.10.3.5), soluble nitrogen (chapter 2.6.1.1), the pH-value (chapter 2.13), Thiobarbituric Acid Index TBI (chapter 2.4), and European Brewery Convention EBC-value (18) were measured according to standardized methods (7, 8) in the accredited laboratories of the Research Center Weihenstephan for Brewing and Food Quality. These brewing attributes characterize the beer and are relevant throughout the brewing process for quality control purposes.

An overview of the values for each sample is given in Supplementary Table S2.

### 2.3. FT-ICR-MS measurements and data processing

The brewing process samples were all subjected to solid phase extraction (SPE) prior to injection into the FT-ICR-MS system. The SPE attributes are given in Supplementary Table S3. The eluate was centrifuged and the supernatant was used for metabolite profiling on a Bruker solariX Ion Cyclotron Resonance Fourier Transform Mass Spectrometer (Bruker Daltonics GmbH, Bremen, Germany) equipped with a 12 T superconducting magnet (Magnex Scientific Inc., Yarton, GB) and a APOLO II ESI source (BrukerDaltonics GmbH, Bremen, Germany) operated in negative ionization mode. The sample order was randomized. Reagents, measurement and data processing attributes were chosen as reported earlier (14, 15). Quality control of the measurement batch was performed by a pooled QC-sample injected in triplicate in the beginning of the batch (QC) and after every 12 samples (QC_A to QC_G). Space charge effects occurring inside the ICR cell were compensated by internal calibration using a particle swarm optimization algorithm based on an in-house calibration list of 2,500 exact masses highly abundant in beer samples (19). The mass resolving power was stable at 400,000 at *m/z* 400 and 87% of all detected monoisotopic signals could be assigned to a molecular formula within an average mass error range of ±0.2 ppm and signal-to-noise ratio of 6. Signals occurring in at least 2 out of 3 replicates were considered and intensity values were averaged. In the 10 min measurements, a total of 8,150 signals with unique unambiguous molecular formulas in the CHNOSP chemical space were obtained within the samples of the two brewing lines and the mass range of *m/z* 120 to 1,000.

### 2.4. Statistical data treatment

For statistical analysis, data pre-treatment included zero-filling, normalization and scaling (Supplementary Table S4). Unsupervised statistics, namely Hierarchical Cluster Analysis (HCA) and principal component analysis (PCA), were performed on FT-ICR-MS data to verify the consistent measurement quality (QC samples) and highlight the process steps that decisively influence the molecular complexity of the beer production. HCA was performed using the complete linkage and Euclidean distance algorithms. For PCA, 95% confidence intervals for various meta data attributes (Supplementary Table S5) were calculated and visualized as ellipses. The Hotelling’s T 2 test (95%) was applied to prohibit the influence of strong outliers on the models.

A correlation matrix (Pearson’s correlation) was used on the beer attributes to find a suitable merging of co-varying attributes to further correlate with FT-ICR-MS molecular data. Brewing attributes with Pearson’s correlation higher than 0.8 were combined. Correlation of the brewing attributes and molecular data was performed by orthogonal projection to latent structures (OPLS) (20) supervised multivariate statistics. The goodness of the fit and prediction were evaluated with the R2Y and Q2 values, respectively. To exclude possible overfitting and provide robust estimates of model performance, we provide the value of p of the 7-fold Cross-Validation Analysis of Variance (CV-ANOVA). High values for the quality of prediction (Q2) in the range of the goodness of the fit (R2Y) and CV-ANOVA *p*-values far lower than 0.01 for the comparison of between-class against within-class variance certified the significance of the models and excluded possible overfitting (21–23). The score values of the brewing samples and statistical attributes of the OPLS-DA model of Pilsner against Munich malt samples can be found in Supplementary Table S5. Scores and statistical values of the OPLS models of FT-ICR-MS data correlated with measured brewing attributes as the y-variable are listed in Supplementary Table S6. Compositions exceeding a Variable Influence on Projection (VIP) value of 1.5 were considered significant (24).

### 2.5. Data visualization

FT-ICR-MS compositional information was visualized in van Krevelen diagrams. By plotting H/C versus O/C atomic ratio of the respective molecular formulas, it is possible to tentatively classify metabolites. Characteristic areas for molecular classes reflect their compositional nature and biochemical origin (13, 25). Molecular patterns of co-varying signals can be revealed (15, 16).

## 3. Results and discussion

### 3.1. The compositional space of brewing

The compositional space of two brewing series were analyzed by direct infusion FT-ICR-MS in biological triplicates. They only differ in the type of malt used. The difference between the two brewing series stems from the utilization of distinct malt types—pale Pilsner malt and darker Munich malt, respectively—both derived from the same raw barley source. Thus, both the molecular similarities of the brewing process steps and the specific differences associated with the way of malting can be comprehended and traced.

Only mass signals detected in at least two of the three biological replicates were considered. Within the two brewing series, a total of 9,370 accurate *m/z* signals were found. In a network calculation approach (19), a unique and unambiguous molecular formula could be assigned to 8,163 (87%) of the accurate masses. The crucial advantage of this annotation approach lies in its ability to cover numerous diverse elemental compositions. The dataset revealed molecular compositions in the C_4-54_H_4-72_ N_0-13_O_2-31_S_0-2_P_0-2_ chemical space. Within a single nominal mass, up to 31 molecular formulae could be assigned to detected mass signals (Supplementary Figure S1). This approach allows us to take advantage of the direct infusion technique without discriminating separation procedures. Molecules with a wide variety of physicochemical properties were characterized, ranging from highly polar, oxygenated, and saturated compounds to less polar, heteroatom-poor, and unsaturated compounds (O/C ratio 0.08–2.25 and H/C ratio 0.66–2.5, respectively). The analytical approach allows us to move beyond the mere mass values and describe the brewing process directly at the compositional level.

**Figure 2:**
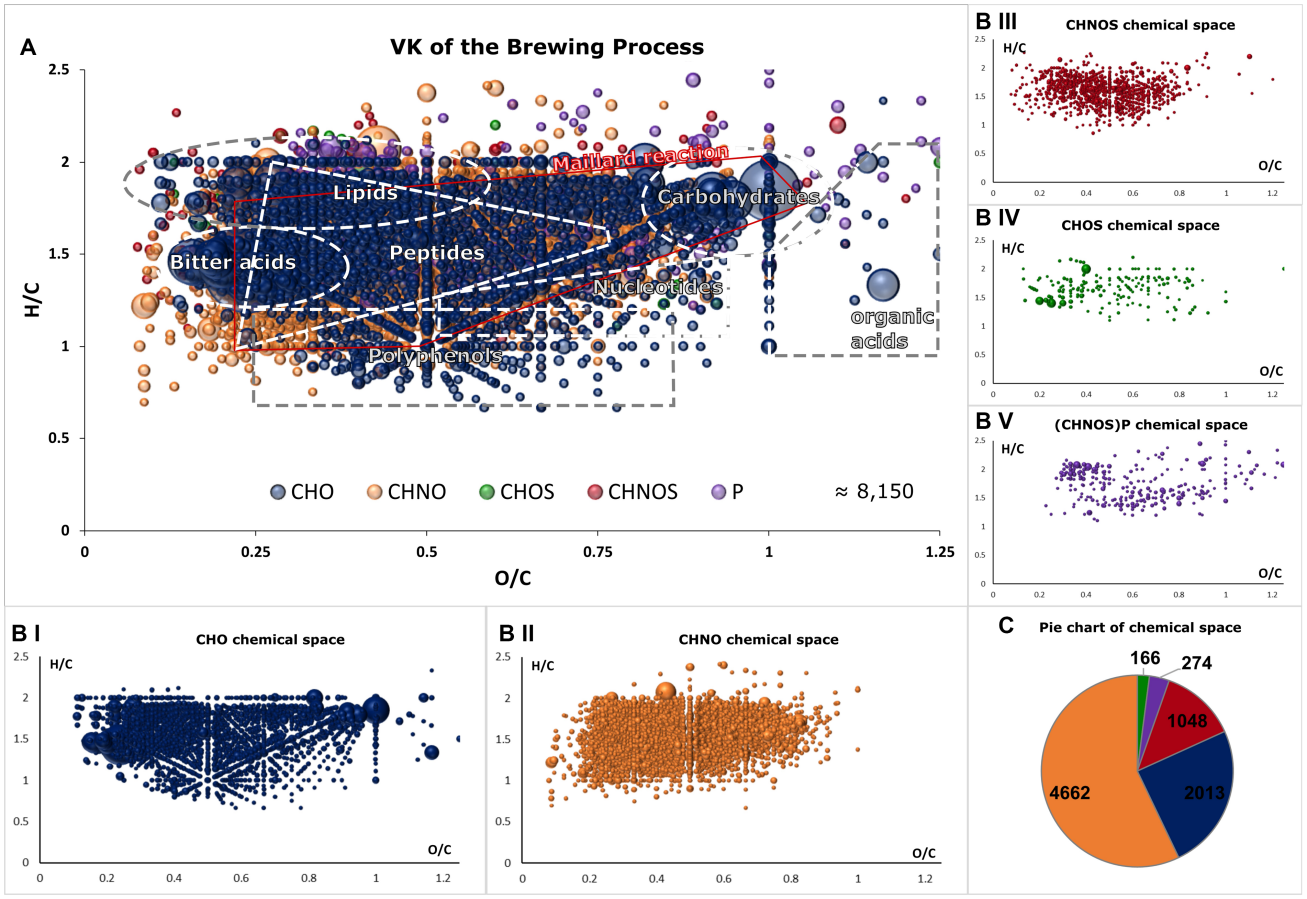
Van Krevelen diagram of all molecular formulae found in the brewing process samples **(A)**. Areas that reflect the range of biosynthetic origin of and allow tentative molecule class annotation are highlighted. Compositional spaces are colored as follows: CHO (blue), CHNO (orange), CHOS (green), CHNOS (red) and (CHNOS)P (violet). The van Krevelen diagram is decomposed into single compositional spaces in **(BI–V)**. One bubble represents one molecular formula and intensities are indicated by their size. In **(C)**, the distributions of the chemical spaces are visualized in a pie chart with corresponding colors.

The *m/z* signals were classified into tentative molecular classes, as visualized in a van Krevelen diagram (Figure 2). The extensive molecular complexity of beer is reflected in the diverse composition of the CHO-chemical space, which encompasses nonpolar lipids, hop terpenophenolics (bitter acids), polyphenols, and highly oxygenated carbohydrates and organic acids (Figures 2A,BI). Nitrogen-containing compounds are predominantly characterized by peptides and Maillard reaction products (MRPs), although some secondary metabolites of plant materials are hidden within (Figure 2BII) (13, 15). When sulfur is involved, for example, through cysteine or methionine, a similar pattern emerges with less pronounced advanced MR products, as extensively described in model systems and the beer Maillard reaction network (Figure 2BIII) (16, 26). CHOS compounds are primarily found as organic sulfate compounds of biochemical origin (Figure 2BIV). Similarly, phosphorus-containing molecules describe both the cascade of Maillard reaction products (from H/C 2 and O/C 1 to H/C < 1 and O/C < 0.2) and phospholipids (Figure 2BV). The findings of previous studies (13), which indicated that the Maillard reaction—particularly the resulting CHNO compounds—account for a significant portion of beer molecules, are confirmed by the proportion of the CHNO space being 57% [CHO: 25%, CHNOS: 13%, CHOS: 2%, (CHNOS)P: 3%] (Figure 2C). The DI-FT-ICR-MS analytical approach enables us to visualize, map, and sort the complex composition of beer and its brewing process within a 10-minute measurement. The comprehensive view at the molecular level provides valuable insights for further statistical analyses.

### 3.2. The molecular evolution of the brewing process

#### 3.2.1. Process steps most impactful on the molecular scale

**Figure 3:**
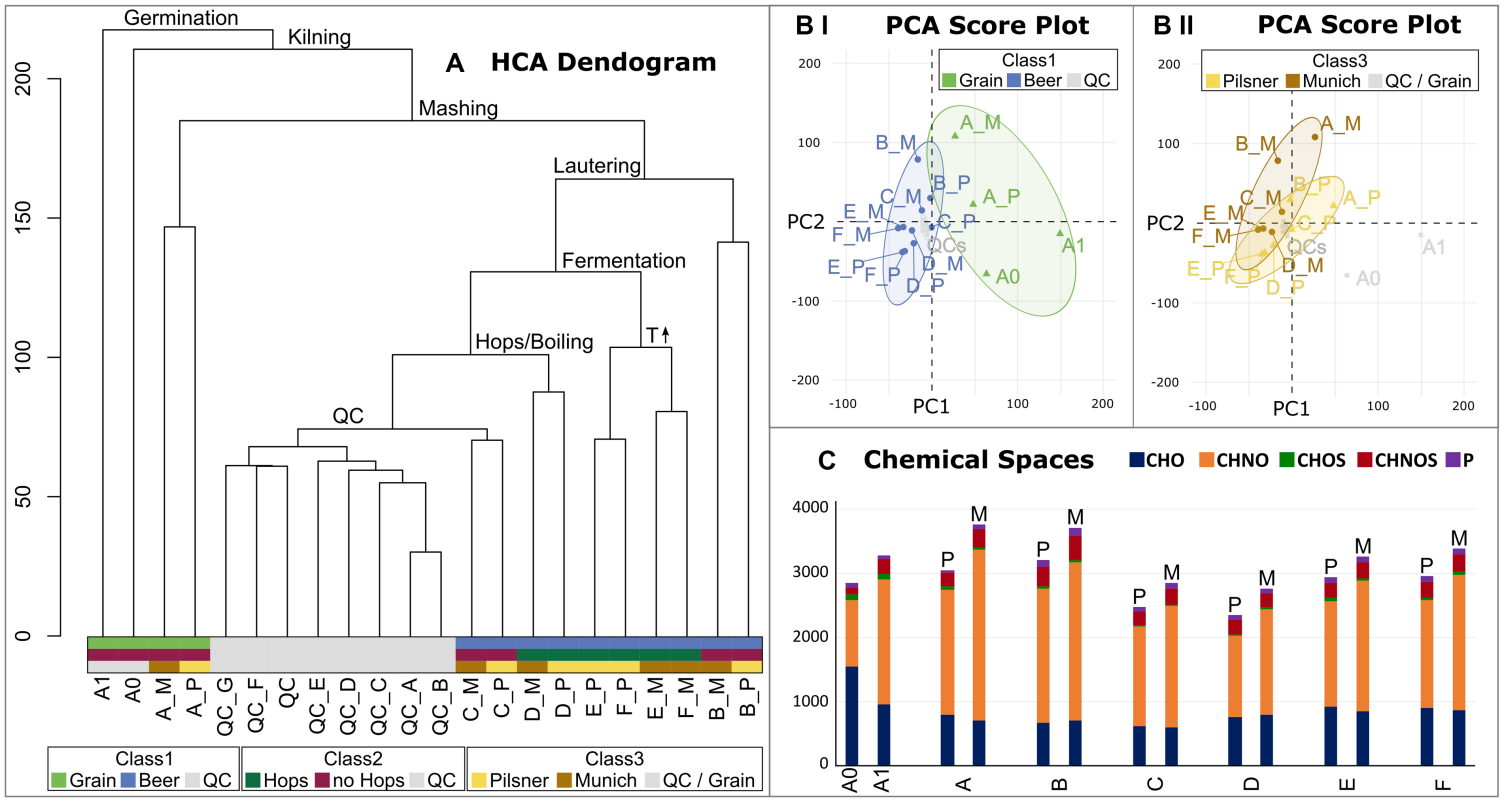
Hierarchical Cluster Analysis (HCA) of the brewing process samples and respective pooled QCs **(A)**. The crucial process steps that explain the branching are given. The QC samples cluster together. The final sample clusters are colored with respect to the sample’s classes (Class1 Grain vs. Beer vs. QC; Class2 Hops vs. no Hops vs. QC; Class3 Pilsner vs. Munich vs. QC/Grain). Score plots of the Principal Component Analysis (PCA) are colored with respect to the sample’s classes (Class1 in **BI**; Class3 in **BII**). 95% confidence intervals are drawn. The distribution of chemical spaces throughout the brewing process is shown in **(C)**. Color code (Figure 2).

The molecular data were subsequently statistically analyzed with regard to the individual process steps and their decisive influence on the beer metabolome. In addition to the brewing process samples, pooled-QC samples were included to statistically validate the consistent quality of the spectra. Hierarchical Cluster Analysis (HCA) structured the samples based on the similarity of their molecular signatures, revealing a distinct cluster of QC measurements (Figure 3A). As suggested by the van Krevelen diagram of the barley grain itself (Supplementary Figure S2) and confirmed by HCA, the most influential process step, from the barley grain to the finished beer, lies in the germination and production of green malt (A1, Figure 1). From here, the brewing process can be traced through the HCA dendrogram, identifying the most influential steps. The completion of malting, i.e., kilning at 80°C (A_P Pilsner) and 100°C (A_M Munich), respectively, separates the malting samples from the samples of the actual brewing process. Interestingly, the malts that were mashed isothermally at 20°C (extract of A_P and A_M) differ significantly from the actual mashing process samples (B). The temperature gradient employed during mashing induces enzymatic processes that show a great impact. The subsequent lautering, although seemingly a filtration and dilution process, points to the crucial commonality of the following process samples (C–F). Having followed the brewing process from germination to lautering, fermentation now takes center stage when considering the most crucial steps of further processing. Alcoholic fermentation differentiates the samples before (C–D) and after the addition of yeast (*S. pastorianus*) (E–F). Evidently, fermentation has a greater impact on the beer metabolome than boiling and even the addition of hops (D). The final steps of bottling and aging show little change in terms of the molecular complexity of the beer. In this final stage, notably, the similarity within the beer lines (E_P, F_P and E_M, F_M) and thus the difference in roast levels is greater than the commonality of aging.

This finding is supported by Principal Component Analysis. The PCA attributes a greater influence on the molecular diversity of brewing to the individual process steps, particularly those related to grain processing (Figure 3BI), than to the distinction between beers brewed with Pilsner or Munich malt (Figure 3BII). Therefore, there are intrinsic molecular signatures of the individual process steps that are independent of the roast level and are universally and reproducibly observed in replicates. These signatures will be further examined in the following.

#### 3.2.2. The brewing process on a compositional level

**Figure 4:**
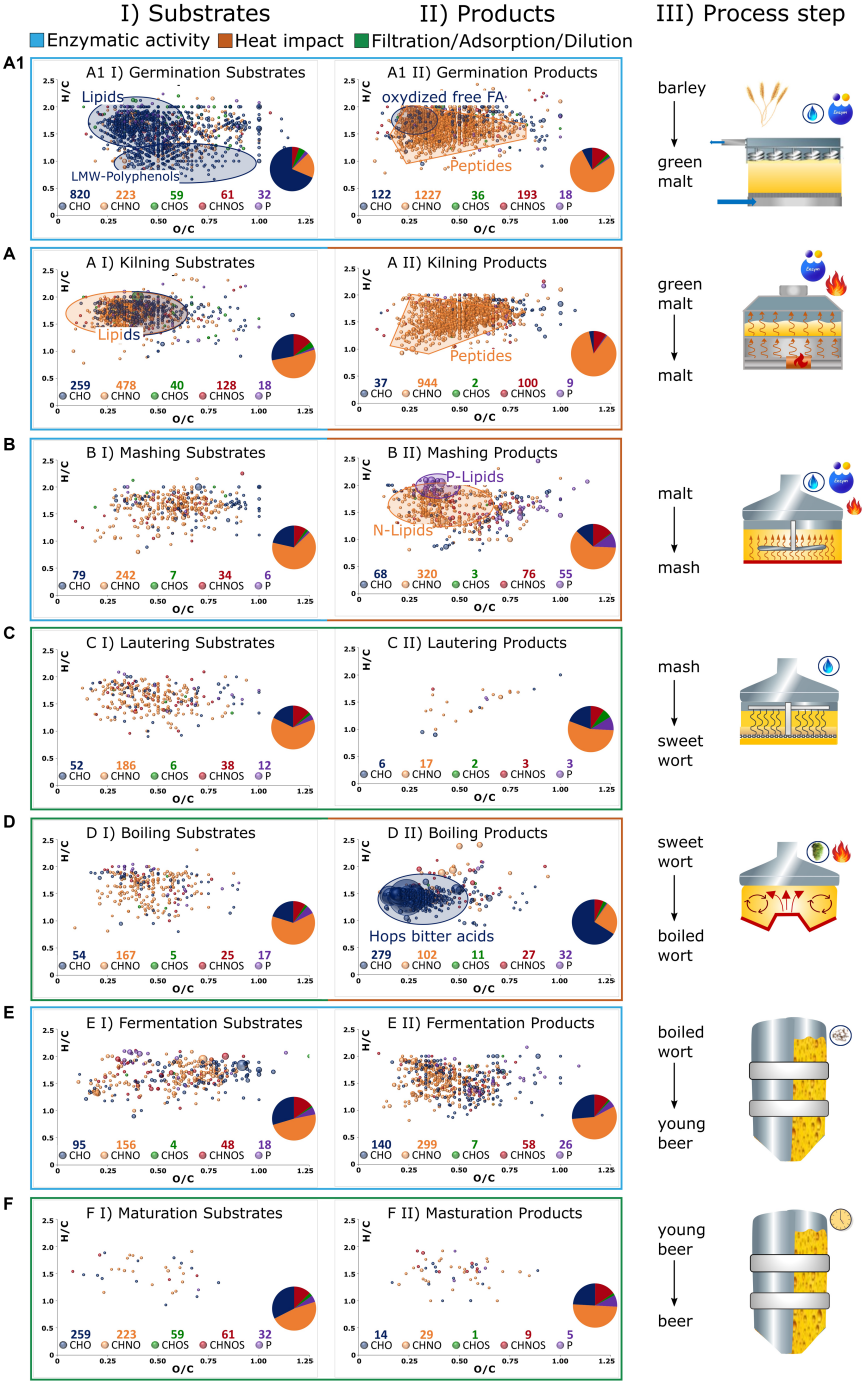
The brewing process is broken down into germination **(A1)**, kilning **(A)**, mashing **(B)**, lautering **(C)**, wort boiling **(D)**, fermentation **(E),** and maturation/filtration **(F)** as graphically described in III. Compositions that get lost during the process are depicted in van Krevelens in I (Substrates). Those that are formed are depicted in II (Products). Compositions that have the same behavior in both Pilsner and Munich beer are considered only. Color codes of the bubbles and sizes (Figure 2). The number of compounds of respective chemical spaces are given below the legend and depicted in a pie chart.

As previously indicated, comprehensive FT-ICR-MS analysis is capable of revealing the dynamic biochemical system of beer brewing based on thousands of molecules. The molecular profile of each sample, both in the Pilsner and Munich series, is visualized as a van Krevelen diagram in Supplementary Figure S2. Although some process steps result in an apparent change in the compositional space, some of the fundamental changes are hidden within the fingerprint of the molecular data. To illustrate process specific imprints, Figure 4 displays the van Krevelen diagrams of those compositions that are consistently degraded in the respective process steps of the Pilsner and Munich brewing lines (I), or newly formed (II).

Germination induces the release and activation of lipases (27). This ultimately leads to the hydrolysis of hundreds of lipids, predominantly triacylglycerides (Figure 4A1I). In conjunction with lipoxygenase activities, it results in the formation of more unsaturated, oxygenated fragments such as an oxo-C18:2 monoglyceride [C_22_H_32_O_5_] (Figure 4A1II). During germination, the polyphenols of the barley grain are deconjugated and made accessible. Their oxidation, involving the introduction of keto groups, ultimately leads to larger polymer structures that exceed the mass range of metabolomic analysis (28). While the decline of numerous monomeric phenolic structures like caffeic acid [C_9_H_8_O_4_], galloyl-glucoside [C_13_H_16_O_10_], or galloylquinic acid [C_14_H_16_O_10_] can be traced (Figure 4A1I), the increased presence of higher molecular weight polyphenols cannot be observed by the metabolomics approach being limited to 100–1,000 Da. By contrast, changes in grain composition induced by proteolytic enzymes exhibit a different pattern. The degradation of proteins generates hundreds of new amino components, most likely peptides and their derivatives (Figure 4A1II). An integrated complementary proteomics approach would certainly serve as a valuable tool in elucidating the underlying mechanisms governing these processes (29, 30). In the convoluted diagram, the presence of several potential secondary metabolites such as gibberellins [C_25_H_34_O_10_], [C_25_H_36_O_12_], and hordatines [C_29_H_40_N_8_O_6_] can also be tentatively identified. Despite the strict criterion that these molecules were not detected before (II) or after (I) the respective process step in both brewing lines, hundreds of characteristic metabolite signals were found to be specific. Nonetheless, it should be noted that as an intrinsic tenet of instrumental analytics, the absence of a molecule signal merely signifies a concentration lower than what is detectable. The presence of 1,195 degraded compositions and 1,596 newly formed compositions within the metabolic mass range substantiate the assertion made by unsupervised statistics, highlighting the central role of germination as the most critical step in chemical transformation.

In the initial stage of drying, both growth and enzymatic reactions continue similarly to germination. When the enzyme activity is either deactivated due to low water content or inhibited by high temperatures, the reactions of amino components and carbonyls predominate, indicating the occurrence of the Maillard reaction. Both phases are reflected in the molecular signature of kilning, encompassing hundreds of compounds. The degradation of lipids progresses, with nitrogen-containing lipids, such as amino acid conjugates or others, being significantly involved (Figure 4AI). The resulting compounds, predominantly distributed in the CHNO chemical space (944/84%) and CHNOS chemical space (100/9%), can be attributed to protein degradation and the onset of the Maillard reaction (Figure 4AII). The compositional space alone does not clearly distinguish these molecular processes from each other. Furthermore, our approach focuses solely on heat-induced processes that occur at both the moderate malting temperatures employed for Pilsner malt and the higher temperatures used for Munich malt. Therefore, extensive Maillard reactions are not expected. However, only a quarter of the malt-specific compounds can be explained by calculated pure peptide masses, indicating the likelihood of derivatization reactions. For these reasons, the Maillard reaction cascade will be further examined in a more specific manner in the following section (3.2.3).

During the mashing process, enzymatic degradation continues and is intensified through a well-designed temperature gradient. The metabolomic changes that are analytically visible primarily involve protein degradation, as well as the oxidation of fatty acids released by lipases during the mashing process [C_20_H_40_O_7_], [C_22_H_40-44_O_6-8_]. Accordingly, the release of phosphatidylcholines/−ethanolamines [C_27_H_44-54_NO_9-12_P] and glycerophospholipids [C_19_H_39_O_7_P], [C_21_H_39-43_O_7_P], [C_24_H_45-47_O_7-9_P] is observed (31) (Figure 4BII). Lautering, as a physical process, does result in adsorption losses and no significant formation of metabolites (Figures 4CI,II). As a commonality, during boiling, an adsorptive loss of compounds is observed due to protein coagulation and the formation of trub (Figure 4DI). Apart from the heat impact, an undoubtedly crucial factor in wort preparation is the addition of hops. The molecular fingerprint of metabolites extracted from hops, visible through FT-ICR-MS analysis, surpasses the traditional hop bitter acids such as (iso)humulone [C_21_H_30_O_5_], (iso)cohumulone [C_20_H_28_O_5_], (iso)lupulone [C_26_H_38_O_4_], and (iso)colupulone [C_25_H_36_O_4_] by orders of magnitude. Particularly, highly oxygenated compounds such as [C_20_H_28_O_7_], [C_21_H_30_O_7_], [C_25_H_36_O_8_], or [C_26_H_38_O_8_] contribute to a total of over 250 derivatives of hop bitter acids (Figure 4DII). The redox chemistry of this compound class’s molecular network, which increases in complexity as the brewing process progresses, has been described in more detail in previous studies (13, 14). Newly introduced polyphenol compositions, extending beyond those derived from malt, manifest predominantly as glucosides (32).

The process of fermentation embodies a distinct and intricate biochemical process that cannot be adequately elucidated solely based on molecular compositions. The nearly constant distribution of chemical spaces suggests the conversion of substrates into products of the same molecular class (Figure 4E). Furthermore, notable observations include nitrogen-containing, saturated, and minimally oxygenated compounds, which have been previously identified as specific to bottom fermentation (14). An exact mass search within the LipidBlast (33) database suggests the presence of hexose-ceramide lipid derivatives. However, to further identify these unknown compounds and understand the underlying biochemical processes, complementary analytical techniques or a comprehensive molecular networking and mass difference enrichment approach are required beyond chemical compositions (34). The maturation processes, as reflected in the non-volatile compound mass range, exhibit minimal overlapping changes (Figure 4F). Thus, the assertion made by unsupervised statistics, emphasizing the persistent influence of different malting methods (Pilsner versus Munich) over maturation-related changes, can be practically confirmed.

#### 3.2.3. Evolution of Maillard reaction products

**Figure 5:**
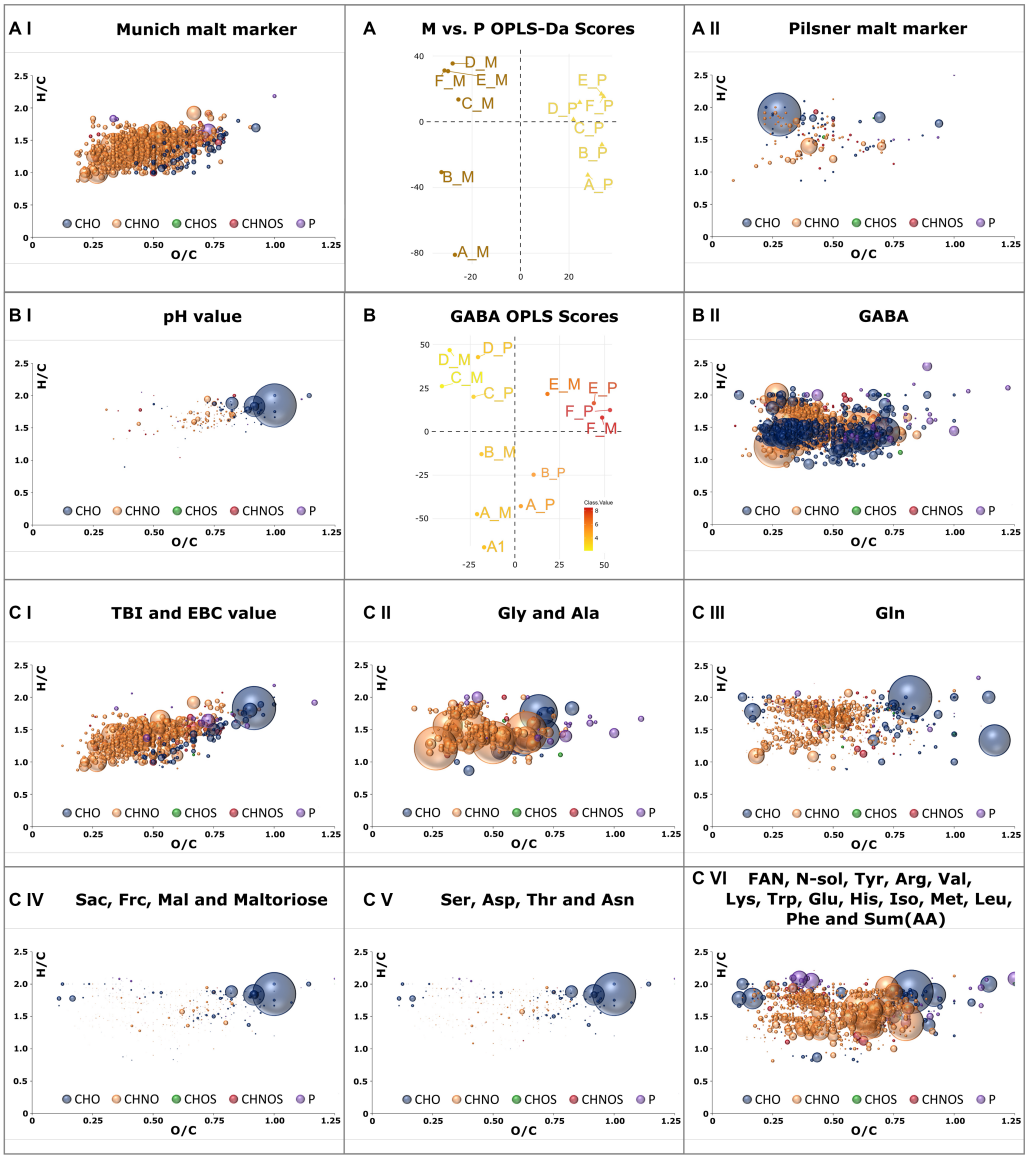
OPLS-DA model of all samples brewed with Munich malt against those brewed with Pilsner malt **(A)**. Van Krevelen diagrams of marker compositions that exceed a Variable Importance in Projection (VIP) value greater 1.5 are plotted for Munich malt **(AI)** and Pilsner malt **(AII)**. OPLS model of FT-ICR-MS data with pH-value as y-variable **(B)**. Positive correlating compositions with VIP values greater than 1.5 are plotted in a van Krevelen diagram in **(BI)**. Those negatively correlating are plotted in **(BII)** as positively correlating with the GABA concentration. Compositions positively correlating with the TBI **(CI)**, Gly concentration **(CII)**, Gln concentration **(CIII)**, Sac concentration **(CIV)**, Ser concentration **(CVI)**, and FAN concentration **(CVI)** and thus strongly correlating with the other attributes given in the plots’ caption are plotted as VK diagrams **(C)**. Color code and size (Figure 2).

The distinct Maillard signature specific to Munich malt was extracted by comparing the samples of the Munich against those of the Pilsner brewing series using OPLS-DA (Figure 5A). Compositions with Variable Importance for Projection (VIP) greater than 1.5 were considered significant and are visualized in a van Krevelen diagram. The 135 compounds that are consistently increased in the Pilsner brewing series, including some degraded peptides and heat-labile lipids, do not exhibit a greater intrinsic order and complexity (Figure 5AII). In contrast, there are 736 Maillard reaction products (MRPs) in the Munich beer samples that possess the same characteristics as described in Pieczonka et al. (16). As the molecular mass increases, the DBE equivalents also increase, resulting in a relatively low degree of saturation. The cascade of reactions, which involves water eliminations as an essential building block, ultimately leads from early MRPs (H/C ≈ 2, O/C ≈ 1), also present in Pilsner beer, to unsaturated, minimally oxygenated late MRPs (H/C < 1.5, O/C < 0.5) (Figure 5AI). The reaction between amino compounds and reducing carbonyls ultimately yields a predominance (84%) of the CHNO chemical space.

The evolution of MRPs, starting from malt (Sample A_M) and ending with the finished beer (Sample F_M), is visualized in Supplementary Figure S3. Although the M-unique compositions cover the same chemical space at each process step (Supplementary Figure S2III), they undergo continuous changes, resulting in only 30–60% of MRPs matching before and after a process step. The distribution of chemical spaces remains consistent (CHNO 75–90%, CHO 10–25%) (Supplementary Figure S3). During the brewing process, the number of oxygen atoms annotated in the formulas decreases due to dehydration reactions. As a result, the O/C and H/C ratios steadily decrease, while the DBEs increase accordingly. The number of nitrogen atoms in the structures slightly but visibly increases, indicating an increasing degree of polymerization. These developments can be explained by the simultaneous progression of MRs in both sample lines. The late MRPs replace the earlier reaction products as unique compounds in the Munich malt brewing series.

### 3.3. Correlation of beer attributes and molecular patterns

Using OPLS, molecular signatures correlating with traditional brewing attributes given in Supplementary Table S2 were extracted. Suitable quantitative y-variables were determined through a correlation matrix, combining brewing attributes with a Pearson’s correlation exceeding 0.8 (Supplementary Figure S4). Both brewing series were considered to obtain generalizable and reproducible results. The concentration of γ-amino butyric acid (GABA) and its strongly negatively correlated pH value (35) were investigated through one OPLS analysis (Figure 5B). Additional score plots of other attributes are compiled in Supplementary Figure S5. The pH drop during fermentation impedes GABA formation, tentatively associated with beneficial health effects (36), by yeast (Supplementary Figure S4BI).

The intrinsic nature of molecular profiling is that beyond mere correlations, causality is not necessarily explained. However, the OPLS model of the TBI (37) and EBC-value (18)—two brewing attributes that depict the thermal impact and the color of the beer, respectively—remarkably demonstrate causality in the van Krevelen diagram (Figure 5CI). The agreement with the characteristic MRPs found in 3.2.3 is 75%. By including Pilsner samples, earlier precursors such as Amadori products gain significance. A crucial difference between the concentration profiles of Gly and Ala compared to other amino acids is their continuous increase from lautering to the finished beer. The molecular profile of the correlating peptides (Figure 5CII) exhibits a distinctly different composition than that of other amino acids (Figure 5CVI). A separate signature was also identified for glutamine (Figure 5CIII). Glutamine concentrations were strikingly showed significantly higher concentrations in Pilsner brewing line samples. The limitations of the methodology become apparent in the profiles of sugars (Figure 5CIV) and the amino acids Ser, Asp., Thr, and Asn (Figure 5CV). With a Pearson correlation of 0.88, these two van Krevelen diagrams are nearly identical and consequently include sugar and peptide signals.

## 4. Conclusion

In conclusion, the application of FT-ICR-MS analysis in studying the brewing process using Munich and Pilsner malt, derived from the same raw barley, has provided valuable insights into the molecular complexity and diversity inherent in brewing with specific reproducible brewhouse conditions. By systematically sampling key stages from raw barley to finished beer, we were able to annotate over 8,000 molecular compositions, tentatively categorizing them into their respective compound classes. All signals were obtained simultaneously in a 10-min measurement. Our statistical evaluation of the chemical mapping of beer unveiled metabolite signatures, including hundreds of compositions, that reflect significant compositional changes occurring during brewing.

The comprehensive molecular analysis revealed a wealth of information regarding the molecular evolution of brewing on the compositional level, offering a multi-dimensional perspective that complements traditional brewing attributes. This expansive molecular imprint, particularly evident in the Maillard reaction products, underscores the chemical complexity that can be unraveled through holistic high-resolution analytical techniques. Moreover, distinct compositional spaces observed allowed us to differentiate between the Pilsner and Munich brewing approaches on a comprehensive molecular scale.

By augmenting well-established brewing attributes, the FT-ICR-MS analysis contributes to the broader understanding of the brewing process and open research challenges. It emphasizes the importance of comprehending the molecular circumstances that underlie the diverse changes encountered throughout the brewing process.

The aspiration is to extend the test series to encompass beers with additional variations in raw materials or process parameters. Complementary proteomics approaches promise insights into the intricate network of enzymatic reactions involved in brewing. Aroma compound analysis, covering the smallest and volatile molecular fraction, and sensorics could complement a future integrated -omics framework, as well as structure elucidation by Tandem-mass spectrometry or NMR. As technology continues to advance and data integration schemes improve, it is conceivable that comprehensive molecular data will emerge as a fundamental tool for efficient monitoring and guiding of the brewing and other production processes in the future.

## Data availability statement

The raw data supporting the conclusions of this article will be made available by the authors, without undue reservation.

## Author contributions

SP, MZ, FA, and PS-K contributed to conception, design, and administration of the study. MZ, FA, and SP organized and conducted the brewing experiments. SP and PS-K designed the methodology of data analysis. SP performed the measurements, data processing, statistical analysis, and data visualization. PS-K, MR, MZ, and MG provided the infrastructure for measurements, data processing, and experimental brewing. SP wrote the first draft of the manuscript. All authors contributed to manuscript revision, read, and approved the submitted version.

## Conflict of interest

The authors declare that the research was conducted in the absence of any commercial or financial relationships that could be construed as a potential conflict of interest.

## Publisher’s note

All claims expressed in this article are solely those of the authors and do not necessarily represent those of their affiliated organizations, or those of the publisher, the editors and the reviewers. Any product that may be evaluated in this article, or claim that may be made by its manufacturer, is not guaranteed or endorsed by the publisher.
