# Supplementary material for: FT-ICR-MS reveals the molecular imprints of the brewing process
Source: Front Nutr. 2023 Sep 22;10:1243503. doi: 10.3389/fnut.2023.1243503 (PMC10557258; doi:10.3389/fnut.2023.1243503)
Supplement: Supplementary file 1 [file Data_Sheet_1.PDF]

Supplementary information

FT-ICR-MS Reveals the Molecular Imprints of the Brewing Process

Stefan A. Pieczonka[1,2,*], Martin Zarnkow[3], Friedrich Ampenberger[3], Martina Gastl[3], Michael Rychlik[1], Philippe Schmitt-Kopplin[1,2,*]

[1]Analytical Food Chemistry, TUM School of Life Sciences, Technical University of Munich, Freising, Germany
[2]Analytical BioGeoChemistry, Helmholtz Association, Helmholtz Munich, Neuherberg, Germany
[3]Research Center Weihenstephan for Brewing and Food Quality, Technical University of Munich, Freising, Germany

*** Correspondence:**
Stefan A. Pieczonka
stefan.pieczonka@tum.de
Philippe Schmitt-Kopplin
schmitt-kopplin@tum.de

Keywords: Beer, Brewing process, FT-ICR-MS, Multivariate statistics, Metabolomics, Molecular profiles, Maillard reaction

Contents

Supplementary Tables 3

Supplementary Table S1. Detailed parameters of the brewing process. 3

Supplementary Table S2. Samples of the brewing process and respective brewing parameter values as an average of the triplicates. 4

Supplementary Table S3. SPE work-up of the beer samples for FT-ICR-MS analysis. 5

Supplementary Table S4. Data treatment prior to statistical analysis. 5

Supplementary Table S5. Metadata attributes and score values of the brewing samples. Statistical values for the PC and OPLS-DA model Munich again Pilsner. 5

Supplementary Table S6. Scores and statistical values of the OPLS models applied to FT-ICR-MS data with brewing parameters as y-variable. 6

Supplementary Figures 7

Supplementary Figure S1. Excerpt of the FT-ICR-MS mass spectrum of the QC sample at the nominal mass of *m/z* 431. 7

Supplementary Figure S2. Van Krevelen diagrams of each sample within the brewing process from the barley grain to the final beer (A0-F). 8

Supplementary Figure S3. Comparison of compositional parameters of molecules unique to Munich beer samples found in Munich malt and the finished beer (A-F). 9

Supplementary Figure S4. Correlation plot (A) and concentration values of measure brewing parameters (B). 10

Supplementary Figure S5. OPLS models' score plots of the FT-ICR-MS data with beer parameters (A-G) as y-variable. 11

Supplementary Tables

Supplementary Table S1. Detailed parameters of the brewing process.

Process	Parameter			
	Time		Temperature	
Steeping wet	6 h		14 °C	
Steeping dry	22 h		14 °C	
Germination	72 h		14 °C	
Kilning	16 h		50 °C	
	1 h		60 °C	
	1 h		70 °C	
	5 h		80 °C (P) / 100 °C (M)	
	4 h		20 °C	
Malt quality	M		P	
	EBC Color 8.0		EBC Color 3.1	
	Water 3.8 %		Water 4.7 %	
	Extract (water free) 82.4 %		Extract (water free) 83.0 %	
	Protein (water free) 11.0 %		Protein (water free) 10.9%	
	Protein Solubility 44.5 %		Protein Solubility 44.4 %	
	Friability-Brittleness 82 %		Friability-Brittleness 79 %	
	Friability-intact grains 0.50 %		Friability-intact grains 0.44 %	
	FAN (EBC ninhydrin) 142 mg/100g		EBC ninhydrin) 160 mg/ 100g	
	Sol-Nitrogen 0.78 g/100g		Sol-Nitrogen 0.78 g/100g	
	Viscosity at 8.6 % 1.52 mPas		Viscosity at 8.6 % 1.50 mPas	
Milling	Universal-malt mill UM2-1650/50 (Täuber)			
Mashing	Mash liquor 18 liter		Grist load 5.2 kg	
	Time		Temperature	
	5 min		62 °C	
	45 min		65° C	
	45 min		73°C	
	2 min		78°C	
Lautering	water addition: 26 L split in 3 steps (8 L, 8 L, 10 L)			
Boiling	Time		Temperature	
	1 h		100 °C	
	Hops: 35 g *Humulus lupulus* variety Tradition (6.6 % α-acids), aiming at 17 IBU			
Fermentation	Pitching rate 15xe6 cells/ml, determined by Nexcelom Cellometer® Vision			
	Yeast was cropped 4 times by removing via cone			
	Time	Temperature	Extract beginning	Extract end
	19 to 22 days	12.3 °C	10.5 to 11.5	1.8 to 2.7
Maturation	Time to diacetly levels less than 0.1 mg/l*: 19 to 22 days			

*Measured by gas chromatography (Mitteleuropäische Brautechnische Analysenkommission (Mebak®) E.V (Rev. 2020-10.). Methode B-420.21.157. Vicinale Diketone - Headspace. [Online]. Available: https://www.mebak.org/methode/b-420-21-157/vicinale-diketone-headspace/729 [accessed August 2023].)

Supplementary Table S2. Samples of the brewing process and respective brewing parameter values as an average of the triplicates.

Sample	Asp [mg/100ml]	Glu [mg/100ml]	Asn [mg/100ml]	Ser [mg/100ml]	Gln [mg/100ml]	His [mg/100ml]	Gly [mg/100ml]	Thr [mg/100ml]	Ala [mg/100ml]	Arg [mg/100	GABA [mg/100ml]	Trp [mg/100ml]	Val [mg/100ml]	Met [mg/100ml]	Iso [mg/100ml]
A0*	7.07	2.47	1.58	1.95	2.81	0.88	1.23	1.85	3	2.11	4.39	1.17	2.05	0.73	1.18
A1*	6.52	3.05	7.19	3.66	19.69	1.69	0.99	2.72	3.33	0.17	3.8	2.71	5.04	0.96	2.82
A_M*	6.58	4	7.09	5.17	15.31	2.59	2	4.28	7.19	6.71	3.7	5.84	7.95	1.99	4.41
A_P*	8.95	5.04	5.92	7.12	37.63	3.63	3.48	6.7	7.5	7.25	5.02	7.45	9.68	2.65	5.23
B_M	9.91	9.28	17.47	8.07	20.43	6.19	3.29	6.94	12.35	16.5	3.79	11.11	14.44	3.79	8.98
B_P	10.04	10.35	16.06	8.16	38.08	6.87	3.85	7.86	15.5	13.82	5.36	12.4	15.95	4.57	10.31
C_M	5.49	5.2	9.95	4.76	8.24	3.76	1.96	4.05	7.05	9.58	2.14	6.58	8.4	2.25	5.48
C_P	6.28	6.25	10.09	5.76	16.51	4.42	2.4	4.9	9.6	8.72	3.33	7.9	9.97	2.88	6.76
D_M	6.27	5.69	13.07	5.37	3.39	4.21	2.17	4.65	7.93	10.63	2.52	7.41	9.57	2.42	6.05
D_P	6.78	6.68	12.76	6.06	6.38	4.71	2.6	5.24	10.24	9.39	3.68	8.46	10.75	3.03	7.17
E_M	4.99	3.47	2.66	1.38	3.69	2.47	2.66	1.26	11.91	6	6.02	5.65	7.37	1.13	3.28
E_P	5.35	4.13	3.29	1.93	4.84	3.11	2.98	1.54	12.93	6.83	7.64	6.66	8.79	1.62	4.43
F_M	5.95	4.78	3.99	2.37	5.15	3.39	3.65	1.57	16.38	8.39	8.42	7.83	10.2	1.52	4.52
F_P	6.51	4.98	4.13	2.44	6.23	3.44	3.6	1.97	14.48	7.53	8.43	7.6	10.14	1.8	4.97

Sample	Try [mg/100ml]	Phe [mg/100ml]	Leu [mg/100ml]	Lys [mg/100ml]	FAN [mg/100ml]	Sum(AA) [mg/100ml]	Frc [g/l]	Glc [g/l]	Sac [g/l]	Mal [g/l]	Maltotriose [g/l]	pH-v.	EBC-v.	TBI	N-sol. [g/100g]
A0*	1.01	1.42	1.95	1.97	4.99	40.85	0.5	0.5	0.63	0.5	0.33	6.22	2.2	1.89	0.25
A1	1.14	4.79	6.03	2.39	9.8	78.63	0.53	1.15	0.63	0.73	0	6.27	1.53	1.25	0.52
A_M*	3.37	8.05	8.46	3.51	13.13	108.21	1.09	2.66	2.76	3.21	0	5.97	6.6	47.93	0.64
A_P*	3.29	10.25	11.38	4.92	16.43	153.09	1.13	4	2.3	3.17	0	6.13	2.77	11.33	0.57
B_M	5.6	15.49	17.78	8.47	24.6	199.86	1.1	9.33	7.53	87.6	26.07	5.68	17.5	113.83	170.33
B_P	5.94	17.72	21.01	10.29	29.7	234.11	1.13	10.3	6.93	96.27	23.13	5.84	6.75	28.03	166.6
C_M	3.04	9.34	10.58	5.13	13.9	112.97	0.7	5.4	4.47	50.13	15.2	5.81	13	77.2	95.13
C_P	3.4	11.39	13.28	6.66	17.23	140.51	0.63	6.67	4.07	55	12.13	6	6.1	22.23	91.87
D_M	3.68	10.63	11.85	5.74	15.17	123.25	1	5.77	4.9	58.47	16.57	5.68	16.17	97.17	100.57
D_P	3.77	12.24	14.18	6.95	17.37	141.05	0.83	6.37	4.27	58.03	13.37	5.88	7	35.3	93.43
E_M	2.97	6.84	4.52	0.77	9.63	79.04	0.03	0.1	0	0.3	0	4.6	13.83	80	86.87
E_P	3.37	8.41	6.68	2.08	11.9	96.61	0.1	0.13	0	0.4	0.17	4.6	5.38	26.3	79.47
F_M	4.22	9.41	6.37	1.32	13.4	109.43	0	0	0	0.07	0	4.6	14.83	80.77	87.77
F_P	3.83	9.47	7.63	2.56	13.73	111.75	0	0	0	0.17	0	4.61	6.18	27.63	81.47

* The solid samples were extracted using water in an isothermal mash at 20°C for 1 hour, according to the referenced standardized analytical methods.

_M Munich malt brewing series, _P Pilsner malt brewing series

A0 Raw Barley, A1 Green malt, A_M Munich malt, A_P Pilsner malt, B Mash, C Sweet wort, D Boiled wort, E young beer, F Finished beer.

Supplementary Table S3. SPE work-up of the beer samples for FT-ICR-MS analysis.

Cartridge	Bond Elut PPL, 1 mL and 100 mg (Agilent Santa Clara, CA, USA)
conditioning	1000 µL MeOH<br>2x 1000 µL Milli-Q Water + 2 % FA
sample	1000 µL acidified sample (2 % FA)
washing	500 µL Milli-Q Water + 2 % FA
dry vacuum	
elution	2x 500 µL MeOH
Centrifugation	4 min at 12,000 rmp on a Centrifuge 5425 (Eppendorf, Hamburg, GER)

Supplementary Table S4. Data treatment prior to statistical analysis.

Data treatment	Method
zero-filling	For each sample, the minimum intensity of the observed peaks was calculated. The average of all samples` minima minus one standard deviation was chosen as “noise level”. Zero-values inside the data matrix were replaced by random values between “noise level” ±σ.
normalization and scaling	z-score

Supplementary Table S5. Metadata attributes and score values of the brewing samples. Statistical values for the PC and OPLS-DA model Munich again Pilsner.

Sample	Class1	Class2	Class3	Class4	PCA scores			OPLS-DA scores	
					PC1	PC2	PC3	P1	O1
A0	Sample	Grain	no hops	Munich	63.8	-66.1	-112.2	-	-
A1	Sample	Grain	no hops	Pilsner	149.6	-15.4	72.6	-	-
A_M	Sample	Grain	no hops	Grain	27.1	108.1	-43.2	-27.1	-80.8
A_P	Sample	Grain	no hops	Grain	47.9	21.6	-6.7	27.6	-32.1
B_M	Sample	Beer	no hops	Munich	-16.4	79.0	-3.5	-32.8	-30.5
B_P	Sample	Beer	no hops	Pilsner	-1.4	30.0	30.5	33.6	-13.4
C_M	Sample	Beer	no hops	Munich	-11.8	14.2	-6.9	-25.7	13.8
C_P	Sample	Beer	no hops	Pilsner	-7.2	-5.3	5.4	21.9	1.5
D_M	Sample	Beer	hops	Munich	-23.7	-11.4	-2.8	-28.2	35.4
D_P	Sample	Beer	hops	Pilsner	-21.4	-27.0	3.7	24.4	12
E_M	Sample	Beer	hops	Munich	-33.3	-7.4	10.3	-30	31
E_P	Sample	Beer	hops	Pilsner	-30.8	-37.1	24.0	33.3	16.3
F_M	Sample	Beer	hops	Munich	-36.9	-7.8	12.2	-31.4	31.1
F_P	Sample	Beer	hops	Pilsner	-31.5	-37.6	25.1	34.3	15.7
QC_A	QC	QC	QC	QC	-8.2	-11.0	-2.2	-	-
QC_B	QC	QC	QC	QC	-7.4	-13.2	-3.0	-	-
QC_C	QC	QC	QC	QC	-9.6	-3.4	-1.0	-	-
QC_D	QC	QC	QC	QC	-10.2	-2.1	0.6	-	-
QC_E	QC	QC	QC	QC	-11.0	-2.8	0.1	-	-
QC_F	QC	QC	QC	QC	-9.0	-5.6	-2.2	-	-
QC_G	QC	QC	QC	QC	-8.6	-1.8	-1.4	-	-
QC	QC	QC	QC	QC	-10.0	2.1	0.6	-	-
					explained variance			RY2	Q2
					20.08 %	15.74 %	12.42 %	0.983	0.733
					total<br>(5 PCs)	78.54 %		p-value<br>ANOVA	<< 0.01

Supplementary Table S6. Scores and statistical values of the OPLS models applied to FT-ICR-MS data with brewing parameters as y-variable.

Sample	pH vs. GABA		TBI		Gly		Gln		Sac		Ser		FAN	
	P1	O1	P1	O1	P1	O1	P1	O1	P1	O1	P1	O1	P1	O1
A1	-17.14	-66.28	-46.33	-94.38	-68.20	-63.58	17.78	90.88	-29.65	-47.85	-19.03	60.70	-35.19	-75.17
A_M	-21.08	-47.31	-2.46	4.71	-26.89	-4.39	2.65	41.66	-5.39	85.22	6.64	77.11	-12.61	50.82
A_P	3.33	-42.75	-34.08	-1.12	31.90	-55.65	62.84	-6.84	-5.58	18.83	38.59	5.94	5.11	-17.43
B_M	-18.73	-12.77	59.05	-22.89	21.43	-1.22	12.99	0.89	58.68	25.58	47.58	13.04	48.48	33.90
B_P	10.43	-24.65	-22.49	22.81	40.16	-34.95	70.48	-43.15	52.91	-11.57	49.19	-19.06	81.54	-34.11
C_M	-40.50	26.23	24.03	-5.27	-27.74	26.68	-13.24	7.00	24.02	0.95	2.00	3.65	-10.74	33.26
C_P	-22.99	19.92	-21.27	16.75	-12.67	11.59	8.00	-10.54	22.01	-13.40	13.44	-17.08	6.78	4.43
D_M	-36.46	46.88	44.50	-10.33	-21.18	37.87	-33.07	4.88	17.65	-29.72	8.20	-25.99	-6.92	15.45
D_P	-20.69	42.78	-11.63	17.43	-6.87	28.32	-24.61	-3.15	15.52	-38.61	19.13	-44.27	5.95	-5.63
E_M	18.10	21.49	28.64	2.71	-1.50	25.51	-31.53	-11.49	-33.80	16.26	-50.09	4.61	-34.13	20.84
E_P	44.13	16.16	-24.28	32.69	11.24	12.55	-21.39	-25.76	-37.58	-10.34	-40.87	-24.17	-19.76	-12.28
F_M	48.69	8.00	29.98	3.72	31.86	11.85	-30.21	-17.10	-39.51	15.81	-38.91	-6.08	-16.00	4.79
F_P	52.90	12.30	-23.67	33.17	28.46	5.44	-20.67	-27.29	-39.28	-11.14	-35.88	-28.42	-12.50	-18.87
	CV-ANOVA p << 0.01													
	R2Y	Q2	R2Y	Q2	R2Y	Q2	R2Y	Q2	R2Y	Q2	R2Y	Q2	R2Y	Q2
	0.995	0.848	0.996	0.748	0.995	0.561	0.991	0.644	0.988	0.824	0.997	0.777	0.996	0.714

Supplementary Figures

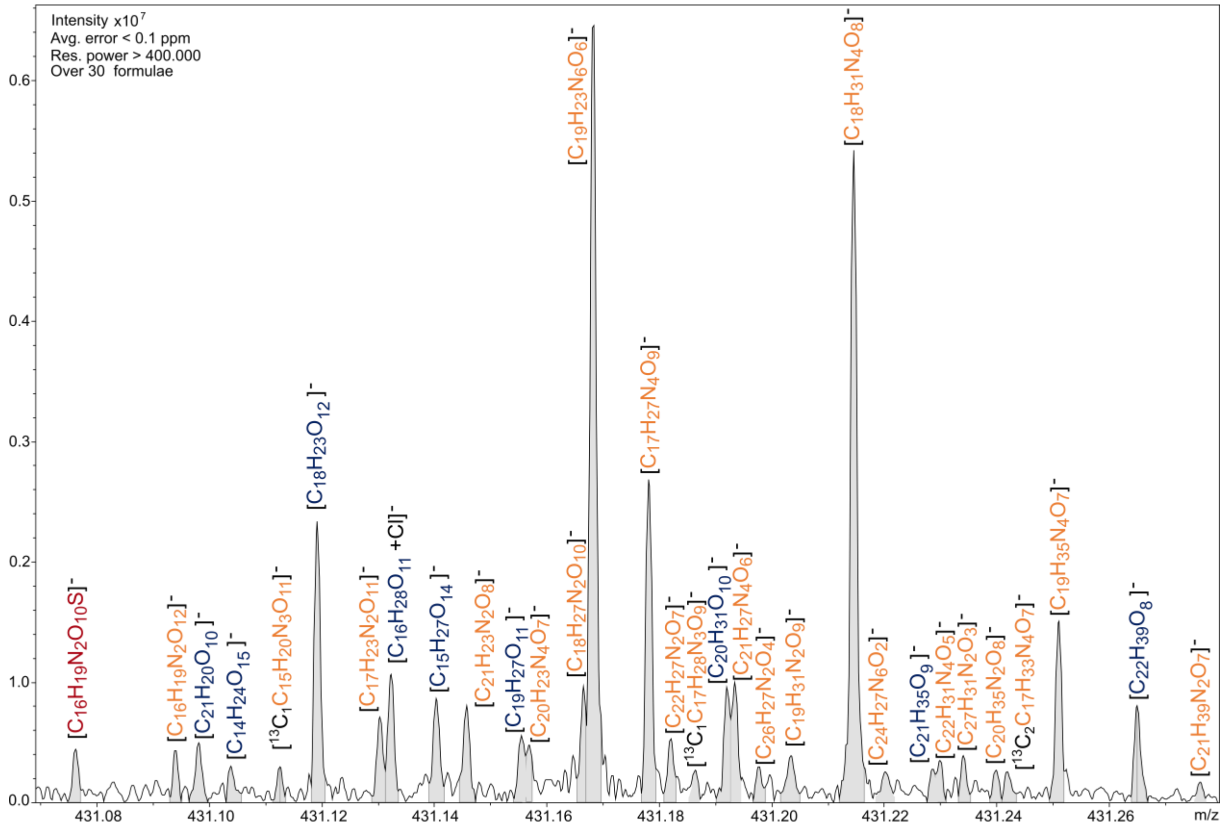

Supplementary Figure S1. Excerpt of the FT-ICR-MS mass spectrum of the QC sample at the nominal mass of *m/z* 431.
Over 30 molecular formulas could be assigned to the mass signals in the CHO (blue), CHNO (orange), and CHNOS (red) chemical space with an average mass error below 0.1 ppm and a resolving power exceeding 400.000.

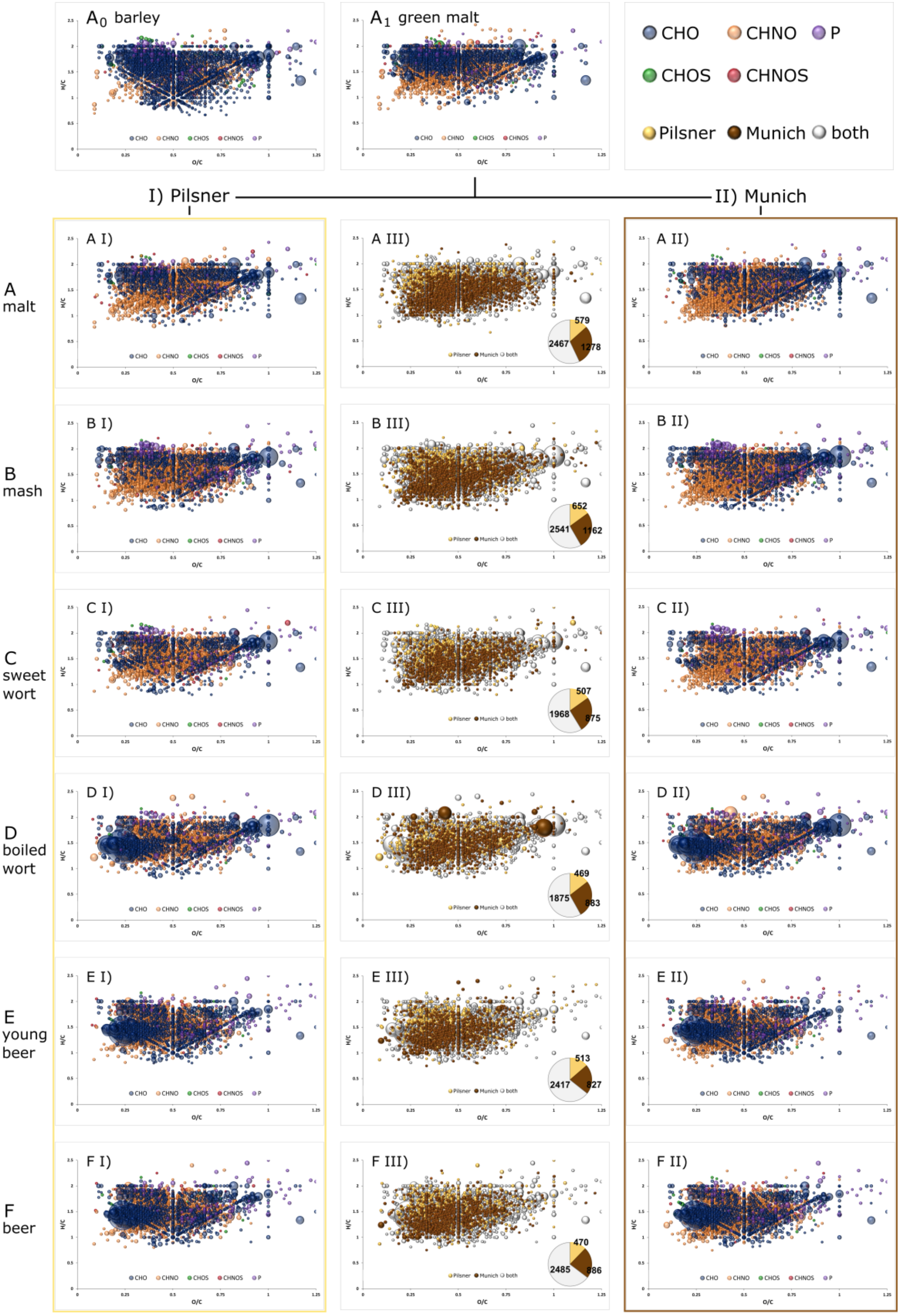

Supplementary Figure S2. Van Krevelen diagrams of each sample within the brewing process from the barley grain to the final beer (A0-F).

After malting, the two brewing lines are separated into the Pilsner malt beer (I) and Munich malt beer (II). The overlap and differences of these brewing lines shown in (III). Color code and bubble size see Figure 2. Van Krevelen diagrams of the molecular changes throughout the brewing process.

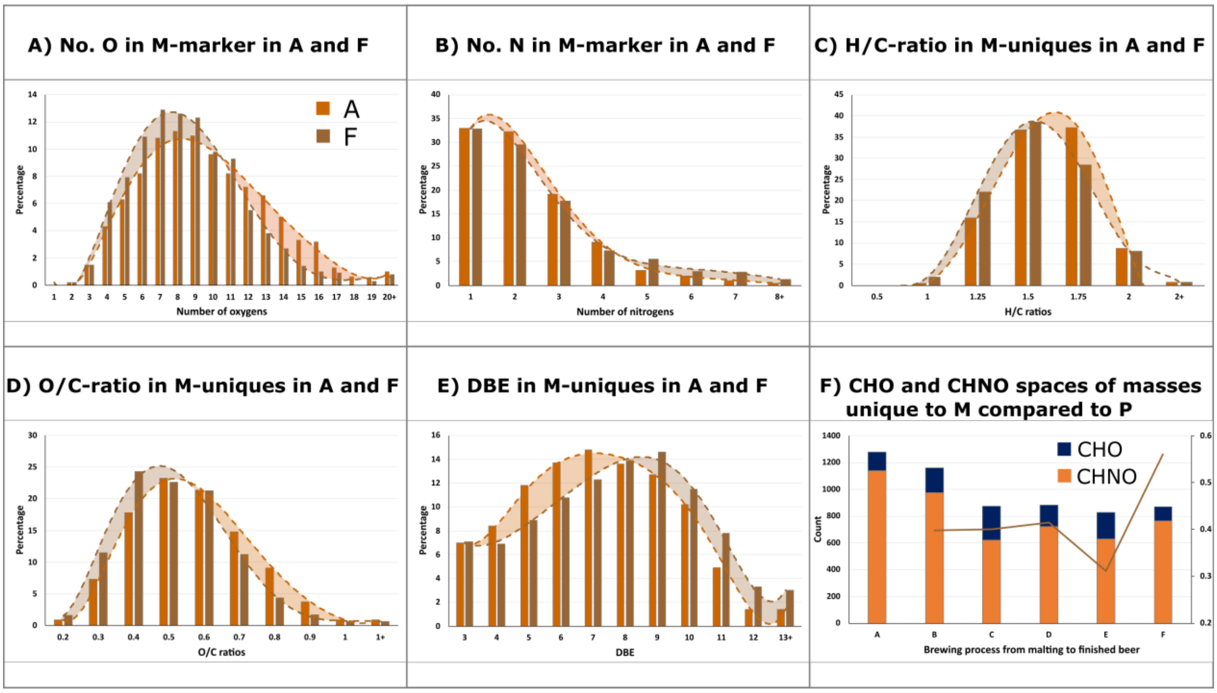

Supplementary Figure S3. Comparison of compositional parameters of molecules unique to Munich beer samples found in Munich malt and the finished beer (A-F).

The number of oxygen in the formula (A), the number of nitrogen (B), the H/C-ratio (C), the O/C-ratio (D), the Double bond equivalents (E) and the CHO and CHNO compositional space are compared (F). In F, the percentage of M molecules congruent with the previous step is shown on the second axis. Color code see Figure 2.

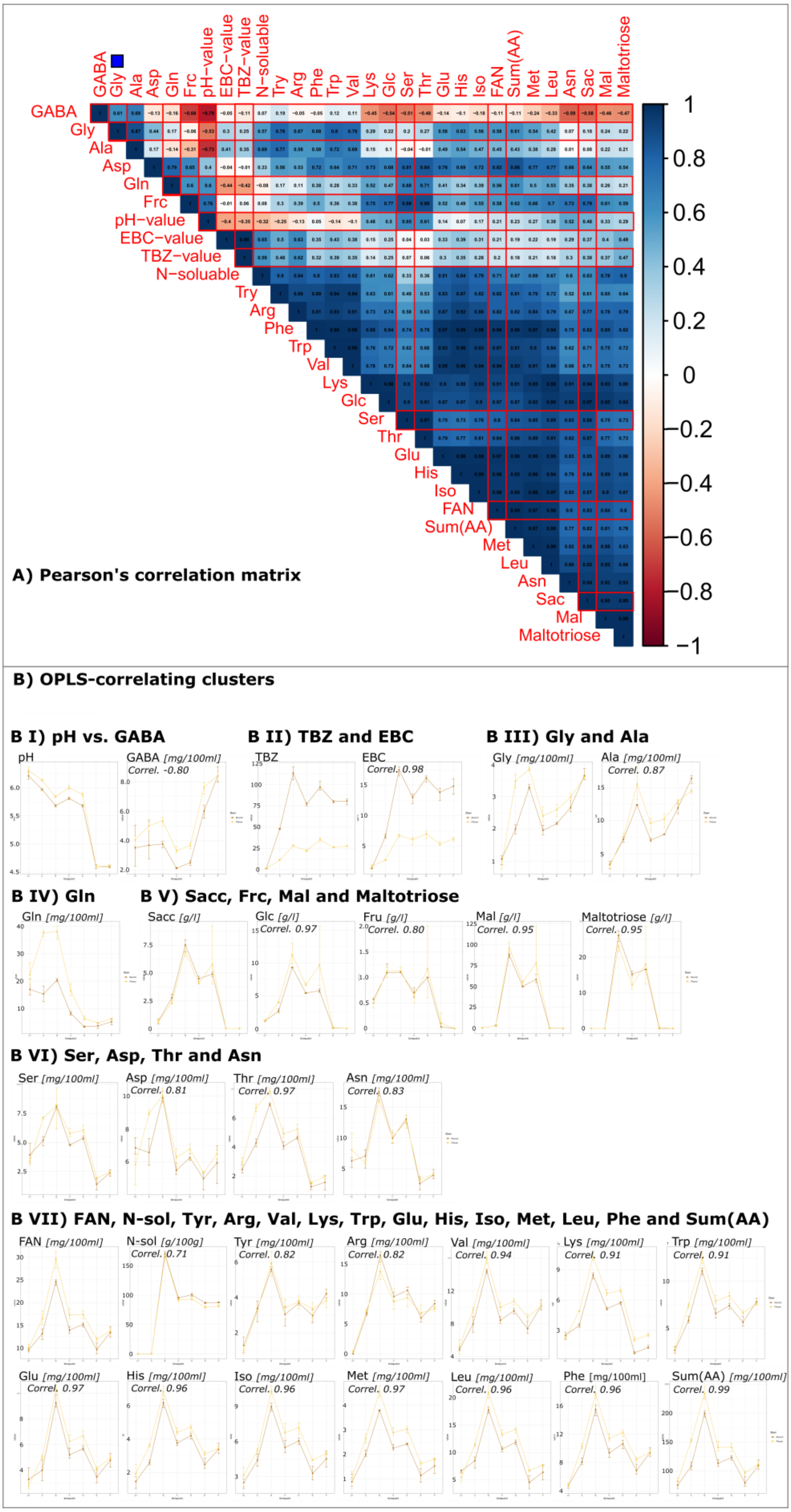

Supplementary Figure S4. Correlation plot (A) and concentration values of measure brewing parameters (B). The correlation plot indicates that investigating correlations of FT-ICR-MS data with 7 different parameters (BI-BVII) will result in a comprehensive approach, because of co-varying of the respective merged parameters. Timepoints order in (B): A1, A, B, C, D, E, F.

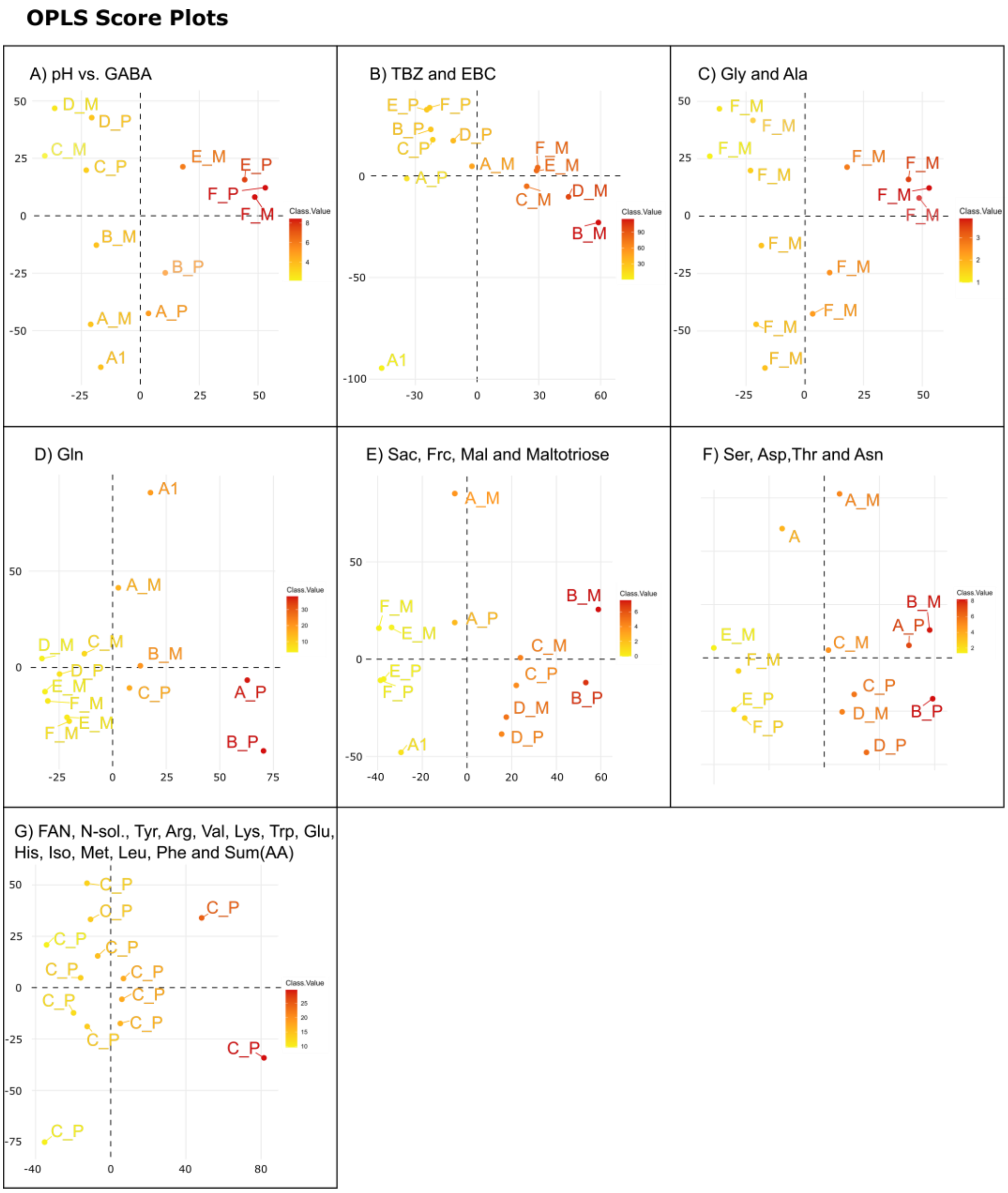

Supplementary Figure S5. OPLS models' score plots of the FT-ICR-MS data with beer parameters (A-G) as y-variable.
The OPLS models describe the metabolite data correlated to the pH (left) against GABA (right) (A), TBI and EBC (B), Gly and Ala (C), Gln (D), Sac, Frc, Mal, Maltotriose (E), Ser, Asp, Thr, Asn (F) and FAN, N-sol., Tyr, Val, Lys, Trp, Glu, His, Iso, Met, Leu, Phe, and sum of amino acids respectively.
